# Utility of SPECT Functional Neuroimaging of Pain

**DOI:** 10.3389/fpsyt.2021.705242

**Published:** 2021-07-29

**Authors:** Mohammed Bermo, Mohammed Saqr, Hunter Hoffman, David Patterson, Sam Sharar, Satoshi Minoshima, David H. Lewis

**Affiliations:** ^1^Virginia Tech Carilion School of Medicine, Roanoke, VA, United States; ^2^School of Computing, University of Eastern Finland, Joensuu Campus, Joensuu, Finland; ^3^EECS - School of Electrical Engineering and Computer Science, Media Technology & Interaction Design, KTH Royal Institute of Technology, Stockholm, Sweden; ^4^University of Washington, Seattle, WA, United States; ^5^The University of Utah, Salt Lake City, UT, United States

**Keywords:** SPECT, pain, ECD, brain, functional imaging

## Abstract

Functional neuroimaging modalities vary in spatial and temporal resolution. One major limitation of most functional neuroimaging modalities is that only neural activation taking place inside the scanner can be imaged. This limitation makes functional neuroimaging in many clinical scenarios extremely difficult or impossible. The most commonly used radiopharmaceutical in Single Photon Emission Tomography (SPECT) functional brain imaging is Technetium 99 m-labeled Ethyl Cysteinate Dimer (ECD). ECD is a lipophilic compound with unique pharmacodynamics. It crosses the blood brain barrier and has high first pass extraction by the neurons proportional to regional brain perfusion at the time of injection. It reaches peak activity in the brain 1 min after injection and is then slowly cleared from the brain following a biexponential mode. This allows for a practical imaging window of 1 or 2 h after injection. In other words, it freezes a snapshot of brain perfusion at the time of injection that is kept and can be imaged later. This unique feature allows for designing functional brain imaging studies that do not require the patient to be inside the scanner at the time of brain activation. Functional brain imaging during severe burn wound care is an example that has been extensively studied using this technique. Not only does SPECT allow for imaging of brain activity under extreme pain conditions in clinical settings, but it also allows for imaging of brain activity modulation in response to analgesic maneuvers whether pharmacologic or non-traditional such as using virtual reality analgesia. Together with its utility in extreme situations, SPECTS is also helpful in investigating brain activation under typical pain conditions such as experimental controlled pain and chronic pain syndromes.

## Introduction

Pain is one of the most challenging clinical entities in medicine. Developments in non-invasive functional brain imaging techniques in the last few decades such as Single Photon Emission Computed Tomography (SPECT), Positron Emission Tomography (PET) and functional Magnetic Resonance Imaging (fMRI) have significantly added to the scientific knowledge about the mechanism of brain processing of pain signals and how it is modulated by different analgesic interventions (1, 2).

Functional brain imaging studies vary in design and investigated target. Receptor imaging studies such as Serotonin and Nicotinic Receptors do not provide temporal information. Fluorodeoxyglucose (FDG) PET utilizes glucose metabolism as a surrogate of neuronal activity. FDG is continuously taken in by the neurons during the uptake time (time between injection and scanning, typically 30–90 min) and does not provide useful temporal information for shorter-lived events such as acute pain or epilepsy. The majority of functional neuroimaging techniques focus on detecting changes in regional blood flow as a surrogate of neuronal activation. fMRI measures/images changes in brain activation while the subject is inside the scanner. fMRI allows real time imaging of brain functional changes during minor or experimental pain experiences with a relatively good temporal resolution. But has limited utility for imaging of severe clinical pain that occurs when the patient is not in the brain scanner, (e.g., during painful medical procedures). It would be difficult or unethical to have the patient inside the scanner during the event. For example, you cannot perform painful wound debridement on a severe burn patient that is in the fMRI borehole (and the patient would have trouble keeping their head very still during the scan, as required to avoid motion artifacts). Similarly, it is difficult to obtain good fMRI brain scans of an epilepsy patient while they are having a seizure due to timing challenges and motion artifacts.

Brain perfusion SPECT using commercially available radiotracers has a unique characteristic that allows freezing an image of brain activation at the time of injection, which can be done in virtually any clinical scenario (e.g., during painful medical procedures conducted outside of the brain scanner, or they can get injected during unpredictable onset epileptic seizures as they are laying in their hospital beds, and the snapshot of brain perfusion at the time of injection is temporarily stored in their brain and can be imaged 1 or 2 h later. This brain activation pattern can be converted into a computer image after the painful event is over, when the patient can be transported and can stay still in the scanner. The concept of freezing an image of pain-related brain activity that can be imaged later is clinically useful in designing functional brain imaging studies where fMRI is not possible due to MRI unfriendly or incompatible clinical circumstances (3), e.g., all virtual reality equipment used in the fMRI scanner must be non-ferrous and non-conductive.

Brain SPECT is a simple technique with minimal stress to the patients. It involves only intravenous administration of a radioisotope during the painful event (e.g., the medical procedure, seizure, or spike in chronic pain) then later laying still in a relatively quiet scanner. Radiation dose varies depending on the radiotracer used and whether an additional low dose CT is used for anatomic localization and attenuation correction to facilitate quantification (4).

Multiple radiotracers have been developed with different purposes; to understand normal brain physiology, to detect static or slowly dynamic brain changes in pathologic conditions, and to investigate brain functional changes at selected time points under natural or experimental pathologic conditions with and without medical interventions. The aim of these studies is to improve our understanding of normal brain function and physiopathological mechanisms of neuropsychiatric diseases. Interpretation of these studies can be either purely qualitative or can provide quantitative/semi-quantitative information (5–7).

## Spect vs. Pet

Compared to PET, SPECT images suffer from limited spatial resolution. This is partially inherent in the physics of the technique, however there have been significant improvements in SPECT spatial resolution with the introduction of high sensitivity solid state detectors such as Cadmium zinc telluride (CTZ) and Cesium Iodide (CsI) as compared to a conventional Anger camera (8).

Temporal resolution is an important factor in designing functional brain studies. Temporal characteristics of functional brain imaging with PET varies with the radiotracer used and the image acquisition technique. Oxygen-15 (^15^O) gas inhalation or labeled water (^15^O-water) infusion has been used as perfusion agents to study experimental brain activation. ^15^O has a very short half-life (~2 min) requiring onsite cyclotron, a complicated imaging setup, and is limited to brain activity that can take place inside the PET scanner only. The most commonly used isotope in PET imaging, Fluorine-18(^18^F) is commercially available with a half-life of about 110 min, eliminating the requirement for an onsite cyclotron. In traditional FDG-PET imaging, using the most popular tracer paralleling glucose metabolism (^18^F-labeled FDG), there is continuous uptake of the tracer by the neurons during the time between radiotracer injection and imaging (the uptake time, typically 30–90 min), which limits its utility to imaging of prolonged brain activity experiences such as interictal imaging of epilepsy, prolonged pain, or placing the patient into a predesigned activation status such as virtual reality (9), walking (10), or prolonged olfactory stimulation (11) during the uptake time. Ripp et al. (12) tested dual time point acquisition of baseline brain metabolism and metabolism with predesigned activation after single FDG injection. There have been recent reports for redesigned FDG-PET functional brain imaging studies with constant infusion of the radiotracer while the patient is inside the scanner and acquiring dynamic images, a technique called functional PET (fPET) (13–15). fPET might gain popularity with the introduction of new high efficiency total body PET scanners and improved time resolution of the camera. The major limitations of fPET are the radiation dose penalty -compared to fMRI which does not use ionizing radiation- and the “activation in the scanner” requirement (16, 17).

## Spect Radiopharmaceuticals

The two most commonly used radiotracers to evaluate brain perfusion using SPECT are Technetium-99 m (^99m^Tc) labeled Hexamethylpropylene Amine Oxime (HMPAO) and Ethyl Cysteinate Dimer (ECD). Both agents have very similar imaging characteristics, however ECD is more popular due its longer shelf life that is very helpful in designing studies when the patient cannot be inside the scanner at the expected time of brain activation, and more importantly when the time of desired brain activity when radiotracer injection is required cannot be predicted, such as ictal epilepsy or migraine studies.

ECD is a lipophilic compound that moves across the blood-brain barrier efficiently and has a high first pass uptake by a normal brain proportional to regional cerebral blood flow with the maximum peak activity reached within 1–2 min after intravenous injection. No significant further radiotracer uptake by the brain takes place a few minutes after intravenous injection. Once taken in by the neurons, it is rapidly de-esterified to a polar metabolite that does not cross the blood brain barrier back and is retained within the brain. ECD does not undergo redistribution within the brain and the gray/white matter activity ratio remains consistently high within the imaging window as measured from multiple sequential SPECT studies. ECD clearance from the brain is relatively so slow that the intracerebral distribution is almost fixed during the time period required inside the scanner. Clearance of ECD from the brain follows a biexponential mode: 40% percent of the brain activity is cleared with a biological half-life of 1.3 h while the remaining activity is cleared slowly with a biological half-life of 42.3 h. There is an additional exponential decay of radiotracer activity with a physical half-life of 6 h. Blood pool activity is cleared rapidly, resulting in high target to background ratio that leads to good quality images starting shortly after injection. ECD demonstrates rapid clearance from facial muscles and salivary glands, further improving image quality. Rapid lung clearance further reduces background activity and improves the brain to soft tissue ratio. The main route of excretion is through the kidneys with a small fraction cleared through hepatobiliary system. The critical organ is the urinary bladder wall (18–23).

## Image Reconstruction

There is no standardized technique for SPECT image reconstruction and viewing. The most commonly used steps involve normalizing measured activity to global brain activity and spatially registering each individual brain to a standard space to eliminate individual differences in the configuration of the brain. Statistical analysis is then performed via voxel by voxel comparison to a normal database. The two most commonly used software packages for image processing and display are Statistical Parametric Mapping (SPM) and Three-Dimensional Stereotactic Surface Projection (3D-SSP). SPM utilizes the *t*-test for analysis of results in high specificity but low sensitivity. 3D-SSP analyzes blood flow to the brain surface (1, 24).

## Imaging of Pain

Functional brain studies allow non-invasive assessment of regional brain activity, generally using blood flow or metabolism as a surrogate of neuronal activity. Advances in functional brain imaging in the last few decades has provided cumulative knowledge about the central mechanisms involved in perception of pain and modulation of this activity by different pharmacologic and non-pharmacologic analgesic interventions (25–27).

Pain is a basic human sensation and an important warning tool against serious conditions. Pain is generally induced by tissue damage or neural pathway abnormality (neuropathic pain). Pain can be acute, chronic, or episodic. Pain is also a complex experience that does not include merely nociception of a stimulus causing sensory input, but is further modified by genetic factors, cultural and environmental factors, memory, circumstantial expectations, anticipation, emotional background, empathy, alertness, motivation, degree of attention vs. distraction, cognitive interpretation and active attempts at modulation of pain perception. Given the diverse nature of human pains and the difficulty to design a study paradigm that completely accommodates for the emotional, cognitive and sensorimotor changes usually associated with the pain experience, it is expected that not all pain experiments will demonstrate the same pattern of brain activation (28–34).

The full anatomic and physiologic process of pain signal processing is not completely understood. The process involves a large network including cortical and subcortical regions. The regions reported to be most consistently activated in functional brain studies during acute pain include the midbrain, thalamus, hypothalamus, amygdala, anterior cingulate cortex, prefrontal region, insula, orbito-frontal cortex, and primary and secondary somatosensory cortices. The term “pain matrix” is commonly used to refer to these regions collectively. Increased regional blood flow in these regions is correlated with the subjective rating of the painful stimuli. The subjective pain experience is further influenced by contextual cortical modulations and the descending pain modulatory system, which can exert inhibitory control at the dorsal horn of the spinal cord to modulate nociceptive input. In other words, the brain can send signals down to the spinal cord, which reduce (or in some cases increase) the amount of nociceptive signals allowed to travel from the spinal cord to the brain. This control system has a cortical component at the anterior cingulate and prefrontal cortex and subcortical components at certain brainstem nuclei (3, 25, 34–39). A small but important study in two subjects who are “pain-free” due to SCN9A mutation, showed activation of the pain matrix during laboratory mechanical pain applied to the dorsum of their hand, similar to response in 4 normal control subjects (40). Further study in this area of understanding the “pain-matrix” for acute pain is needed.

A pain processing network model by Garcia et al. (29) suggested that pain is processed at three levels, at an unconscious level processed in peri-Rolandic cortex and limbic system receiving afferent spinothalamic pain signals, at an intermediate awareness level processed at fronto-cingulate-parietal networks in addition to the sensorimotor cortices, and at a higher conscious extended level which includes adding input from memories and self-awareness. The comprehensive pain experience processing network appears to include more regions with contribution from other cortical and subcortical cerebral regions such as the brain stem and the cerebellum (3, 29).

## Chronic Pain

Chronic pain syndrome is a difficult clinical entity that is not completely understood. Some studies suggested that dysfunctional coordination between ascending and descending pain pathways plays a major role in the pathophysiology of chronic pain syndrome. Brain activation appears to be different in chronic pain compared to acute pain (25, 41).

Nakamura et al. (4), recruited low back pain patients with no significant abnormalities in the lumbar spine detected during MRI and reported significantly decreased blood flow in the bilateral prefrontal cortex in patients with chronic low back pain compared to patients with acute low back pain. In a controlled study of 12 patients with chronic pain using ECD SPECT, Nakabeppu et al. (41) reported a significant decrease in blood perfusion in the thalamus bilaterally in chronic pain patients compared to their control counterparts. Honda et al. (1) studied 15 chronic pain patients using SPECT and reported reduction in rCBF in several brain areas (e.g., prefrontal area, right orbitofrontal cortex, anterior cingulate gyrus).

## Fibromyalgia (FM)

Several studies have demonstrated the role of ECD brain perfusion SPECT in imaging of brain activity changes in Fibromyalgia (FM) patients before and after therapy (42–50). Chen et al. studied 91 patients with FM and reported reduction in blood flow in the temporoparietal and frontal regions in addition to the thalamus and basal ganglia (42). In a study of fibromyalgia patients, baseline thalamic blood flow was decreased below normal age matched database. Thalamic blood flow improved after electroconvulsive therapy (ECT), this improvement was correlated with subjective reporting of improved level of pain (47).

In a controlled study of 18 hyperalgesic FM female patients, Guedj et al. reported significant hypoperfusion in the somatosensory cortex as well as frontal, cingulate, medial temporal and cerebellar cortices (50). In another study of 20 Fibromyalgia patients, the same group studied the correlation between cerebral blood flow and pain using several self-reported pain measurement as well as depression and anxiety scales. They reported that the clinical severity of the disease was correlated with abnormalities of blood flow (43). The authors suggested that SPECT can guide therapeutic strategies for patients as an objective measure. In two other studies, the same group reported that SPECT predicted analgesic response to ketamine in hyperalgesic FM patients. The authors showed a significant hyperperfusion in midbrain periaqueductal gray in patients reporting reduction in subjective pain after Ketamine (responders) vs. non-responders (49) while non-responders exhibited a significant hypoperfusion in bilateral medial frontal gyri (50).

Usui et al. reported rCBF abnormalities in FM patients compared to their control counterparts including decreased perfusion at the left culmen and increased perfusion in the right posterior cingulate, precentral, superior occipital, and middle temporal gyri and right cuneus, and increased perfusion at the left superior and inferior parietal lobules and postcentral gyrus. Furthermore, patients with good response to gabapentin demonstrated significant hypoperfusion in the right medial frontal gyrus, left insula, left inferior frontal gyrus, and left culmen and increased perfusion in the left superior frontal and postcentral gyri while poor responders demonstrated significant decreased perfusion to the left orbital gyrus and hyperperfusion in the right precentral and postcentral, posterior cingulate, and superior temporal gyri, right precuneus, right inferior parietal lobule, and left middle frontal and middle occipital gyri (48).

### Episodic Pain

Ictal perfusion SPECT imaging has been used for presurgical evaluation of epilepsy patients for decades. Subtracting interictal from ictal SPECT perfusion studies and overlying the subtraction results on structural imaging, particularly MRI, is the most accurate functional imaging technique for localizing the seizure onset zone in patient with epilepsy (51, 52). A rare form of seizure is Ictal pain. While epileptic pain is usually associated with other seizure symptoms, sometimes pain is the only symptom of epilepsy. It can be unilateral or bilateral, pain location varies, most commonly in the head and neck or abdomen (53).

The unique characteristic of SPECT tracers makes it feasible to study regional perfusion changes in other episodic pain syndromes such as migraine during the attack (ictal) and between the attacks (interictal). Similar to epilepsy ictal SPECT studies, ictal migraine study setup is challenging and requires patient hospitalization and exposure to potential migraine triggers with a trained nurse available by the patient's side ready to inject the radiotracer as soon as the patient starts to experience the migraine aura. The longer shelf life of the ECD compound compared to HMPAO is very helpful in these situations as the waiting time is unpredictable. Significantly reduced rCBF at the thalamus on SPECT has been reported in two patients with ophthalmoplegic migraine (54) and in a child with hemiplegic migraine (55). It is not clear, however, if the changes in regional blood flow associated at the ictal phase of migraine is related to the primary etiology or is a secondary phenomenon (56).

### Response to Therapy

**Figure 1:**
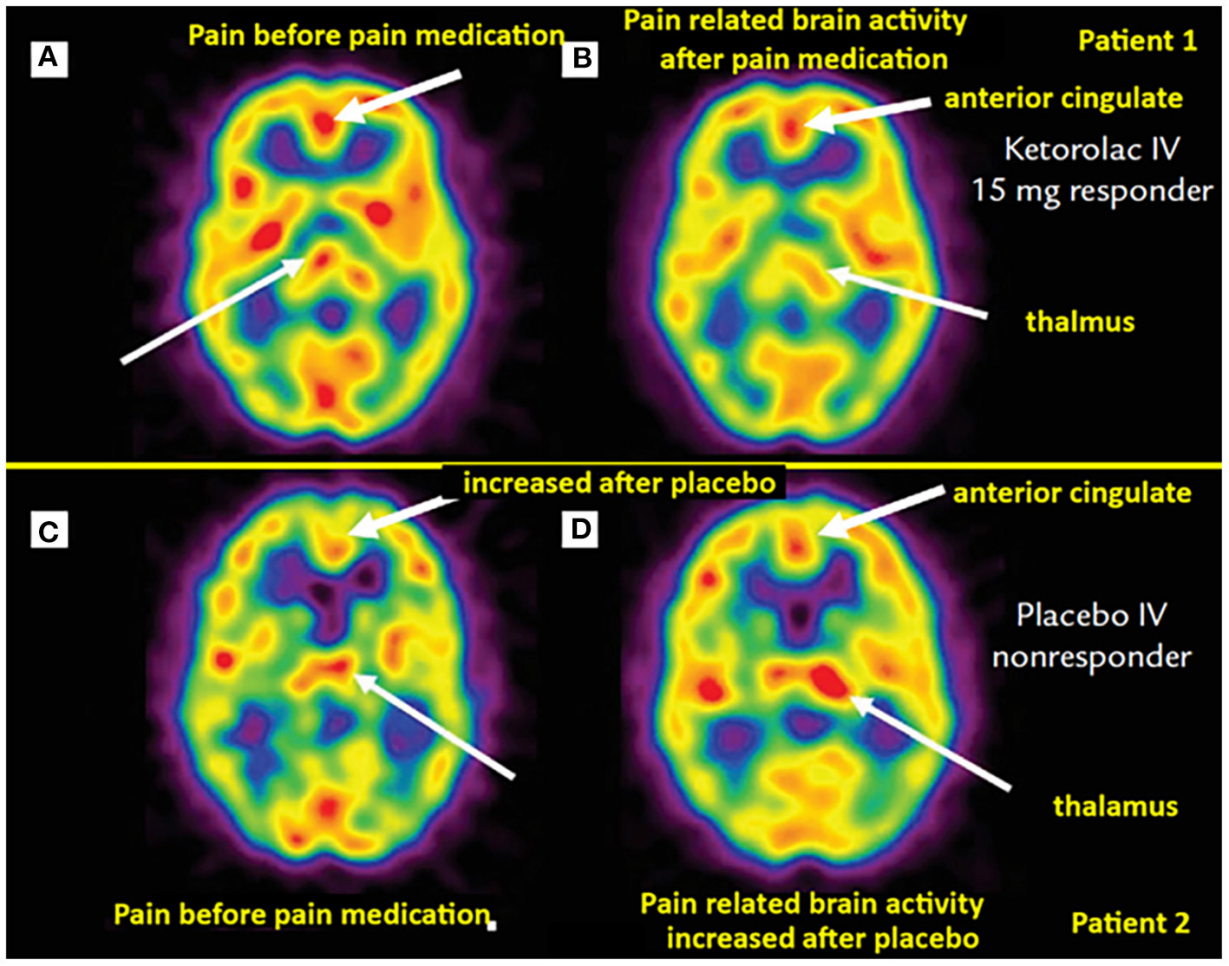
Brain perfusion SPECT of dental pain patients receiving analgesia (top row) vs. placebo (bottom row). **(A)** Asymmetric thalamic activity, more on the right (thin arrow). Post IV ketorolac, the post-interventional scan **(B)** of the same patient exhibits a slight “switch” in thalamic asymmetry, with mildly greater perfusion on the left (thin arrow). Noted also decreased perfusion in the anterior cingulate region with pain relief [thick arrows in **(A)** and **(B)**]. **(C)** Scan from another patient demonstrating mild asymmetric increased activity in the left thalamus (thin arrow). **(D)** Same patient with worsening pain after receiving IV placebo, the scan demonstrates more asymmetrically increased perfusion in the left thalamus (thin arrow). Not also increased perfusion in the anterior cingulate cortex compared to **(C)** (thick arrows). [Images by Newberg et al. (58), reproduced here with permission].

Brain perfusion SPECT has been used to study not only brain activation with pain, but also modulation of this activity under pharmacologic and non-pharmacologic analgesic interventions. In addition to the previously discussed studies demonstrating blood flow changes in response to therapy in FM patients, Trucco et al. (57) demonstrated reversal of perfusion abnormality on brain perfusion SPECT in migraine patients under pharmacologic therapy. Newberg et al. (58) reported asymmetric thalamic blood flow in acute postoperative dental pain, this perfusion abnormality improved in patients receiving successful analgesic treatment (Figure 1). A study of brain perfusion SPECT in the setting of severe clinical pain during burn wound cleaning/debridement demonstrated intense activation of the cerebellum, this activation was reversed in the same patients when using immersive virtual reality analgesia in a different session (59).

A study evaluating the effect of analgesic acupuncture on regional blood flow demonstrated a significant asymmetric uptake in the thalami in pain patients compared to controls. This abnormal thalamic flow was normalized in the post acupuncture therapy scan (60).

Fukui et al. (61) demonstrated normalization of the thalamic hypoperfusion in complex regional pain syndrome patients after ECT. Changes in rCBF have also been reported following deep brain stimulation for chronic pain (62). Tamura et al. studied seven normal subjects and reported a significant correlation between improved subjective pain and rCBF changes measured by SPECT after repetitive transcranial magnetic stimulation on acute pain induced by capsaicin (63).

### Limitations

The brain perfusion SPECT technique has its limitations: the technique utilizes ionizing radiation, mainly from the injected radiotracer and additionally from the optional low dose CT sometimes used for rough localization and attenuation correction. Software fusion of SPECT and MRI images instead provides significantly better anatomical details and avoids the radiation penalty associated with CT. Multiple conditions typically cannot be evaluated during the same session. The ability to repeat the study to investigate multiple variables or time evolution of one variable is also limited due to the irradiation dose, cost, and the complexity of the SPECT procedure. The technique is more complex compared to fMRI as additional steps related to the handling and injection of the radiopharmaceuticals are involved. While temporal characteristics are unique in one aspect, it is still limited, because it is basically a summed 1–2 min of brain activation with less temporal resolution than fMRI. The control study is typically performed on a separate day. Data processing and image display are not standardized. There are also problems related to differences in interpretation of results according to the experience of the radiologist so reproducibility and interobserver agreement is not high (64–67).

In summary, despite these limitations, one of the great advantages of SPECT is that unlike most neuroimaging modalities, with the SPECT technique, the patient does not need to be in the scanner at the time of brain activation. SPECT freezes a snapshot of brain activity at the time of injection that is kept and can be imaged later (after wound care is completed). The SPECT technique allows researchers to measure brain activity in much wider range of clinical settings, increasing the ecological validity of clinical pain research and potentially increasing our understanding of pain-related brain activity during painful medical procedures, and during painful spikes in chronic pain. Additional research and development of brain perfusion SPECT technique is recommended.

## Author Contributions

MB and DL: conception and design. MB and HH: collection and assembly of figures. MB, MS, HH, DP, SS, SM, and DL: manuscript writing and final approval of manuscript. All authors contributed to the article and approved the submitted version.

## Conflict of Interest

The authors declare that the research was conducted in the absence of any commercial or financial relationships that could be construed as a potential conflict of interest.

## Publisher's Note

All claims expressed in this article are solely those of the authors and do not necessarily represent those of their affiliated organizations, or those of the publisher, the editors and the reviewers. Any product that may be evaluated in this article, or claim that may be made by its manufacturer, is not guaranteed or endorsed by the publisher.
